# Supplementary material for: Extending the coherence of spin defects in hBN enables advanced qubit control and quantum sensing
Source: Nat Commun. 2023 Aug 22;14:5089. doi: 10.1038/s41467-023-40473-w (PMC10444786; doi:10.1038/s41467-023-40473-w)
Supplement: Supplementary file 1 — Supplementary Information [file 41467_2023_40473_MOESM1_ESM.pdf]

Supplementary Information to "Extending the coherence of spin defects in hBN enables advanced qubit control and quantum sensing"

Roberto Rizzato*,[1,6] Martin Schalk,[2,3] Stephan Mohr,[1] Jens C. Hermann,[1,3] Joachim P. Leibold,[1,4] Fleming Bruckmaier,[1] Giovanna Salvitti,[1,7] Chenjiang Qian,[2] Peirui Ji,[2] Georgy V. Astakhov,[5] Ulrich Kentsch,[5] Manfred Helm,[5] Andreas V. Stier,[2,3] Jonathan J. Finley,[2,3] and Dominik B. Bucher*[1,3]

[1]*Technical University of Munich, TUM School of Natural Sciences, Department of Chemistry, Lichtenbergstraße 4, Garching bei München, 85748, Germany*
[2]*Walter Schottky Institute, TUM School of Natural Sciences, Am Coulombwall 4, Garching bei München, 85748, Germany*
[3]*Munich Center for Quantum Science and Technology (MCQST), Schellingstr. 4, München, D-80799, Germany*
[4]*Technical University of Munich, TUM School of Natural Sciences, Department of Physics, James-Franck-Str. 1, Garching bei München, 85748, Germany*
[5]*Helmholtz-Zentrum Dresden-Rossendorf, Institute of Ion Beam Physics and Materials Research, Bautzner Landstraße 400, Dresden, 01328, Germany*
[6]*University of Bari, Department of Physics "M. Merlin", Via Amendola 173, 70125 Bari, Italy*
[7]*University of Bologna, Department of Chemistry "G. Ciamician", Via Selmi, 2, 40126 Bologna, Italy*

CONTENTS

Supplementary Note 1: ESEEM modulation 1
Supplementary Note 2: Decoherence curves with a CPMG protocol for N=300, 600, 800, 1000 π-pulses 1
Supplementary Note 3: Fundamentals of sensing RF fields using spin defects in solid-state systems 1
1. MW control of the V_B^- qubits 2
2. Interaction with external RF fields 2
3. Phase accumulation in a spin-echo experiment 2
4. Multipulse dynamical decoupling (pDD) schemes 4
5. Slope and variance detection 4
6. Experimentally probed phase accumulation with V_B^- in hBN 5
7. Phase accumulation in the spinlock experiment 6
8. Coherently Averaged Syncronized readout (CASR) and estimation of sensitivity 6
Supplementary Note 4: Extension of the coherence times by the XY8-N protocol 8
Supplementary Note 5: Estimation of sensitivity and comparison with NV-diamond sensors 8
Supplementary Note 6: Optimizing conditions for sensing RF fields with V_B^- in hBN 9
Supplementary Note 7: Coherent manipulation of the V_B^- dressed-states 10
Supplementary Note 8: Fitting Details 12
1. ODMR spectrum 12
2. T_2 measurement 12
3. Extension of the coherence times 12
4. RF sensing with the XY8-2 pulse sequence 12
5. RF sensing with the spinlock pulse sequence 12
Supplementary Note 9: Raw data 13

References 14

Supplementary Note 1: ESEEM modulation

In the T_2 measurement reported in Figure 1 (h) and in the CPMG experiments in Figure 2 (a), we observe a strong modulation of the time-domain signal reminiscent of the electron spin envelope modulation (ESEEM) observed in other systems when the central electronic spin strongly interacts with proximate nuclei, such as ^{14}N and ^{13}C. Fitting this oscillation with a function $F(t) \propto \cos(2\pi f t)$ yields a frequency of $f \sim 45\,\mathrm{MHz}$, matching the hyperfine coupling of the three equivalent ^{14}N surrounding the defect. A similar effect can also be predicted using a well-known EPR calculation toolbox EasySpin [1] by simulating a two-pulse ESEEM experiment. In Supplementary Figure 1, we compare the decoherence curve obtained with a spin-echo T_2 measurement to the one obtained with EasySpin. In the simulation, three ^{14}N have been considered in the spin system, coupled to the electronic spin with the following hyperfine tensors A_1 = [80 57 47] MHz, A_2 = [46 91 48] MHz, A_3 = [80 57 47] MHz [2, 3]. Further parameters are: spin S = 1; g-factor = 2; zero-field-splitting: D = 3.47 GHz, E = 60 MHz, magnetic field B_0 = 8 mT, MW frequency ν_{MW} = 3.2 GHz, excitation bandwidth Δ_{exc} = 250 MHz, Exp.Sequence=2pESEEM, resolution dt = 0.001 μs. Relaxation parameters: T_1 = 6 μs, T_2 = 80 ns.

Supplementary Note 2: Decoherence curves with a CPMG protocol for N=300, 600, 800, 1000 π-pulses

In Supplementary Figure 2, we display the datasets for the decoherence curves with $N = 300, 600, 800,$ and 1000 π-pulses, enclosed in the dotted box in Figure 2 (a) of the main text. The scaling has been adjusted to allow for a better visualization of the data points and fittings.

Supplementary Note 3: Fundamentals of sensing RF fields using spin defects in solid-state systems

In this study, we employ ensembles of V_B^-centers in hBN as a spin sensor for RF magnetometry. Each V_B^-sensor can be viewed as a qubit with two selectable ground state levels, for instance: $|m_s = 0\rangle$ ($|0\rangle$) and $|m_s = 1\rangle$ ($|1\rangle$). By

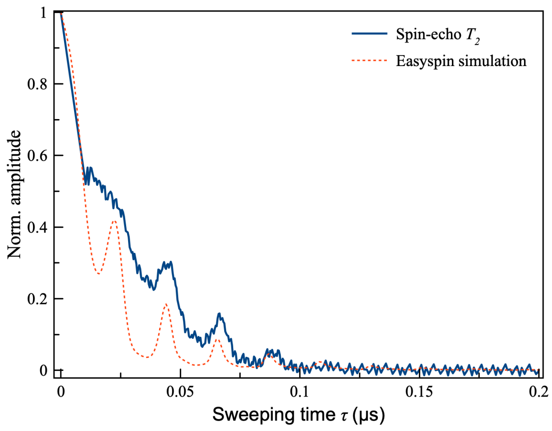

Supplementary Figure 1. A two-pulse ESEEM experiment is simulated with the EasySpin function "saffron" and compared with experimental data.

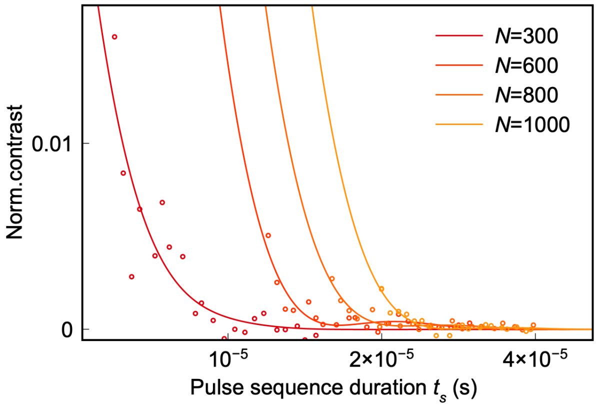

Supplementary Figure 2. Decoherence curves with $N = 300, 600, 800,$ and 1000 π-pulses, enclosed in the dotted box in Figure 2 (a) of the main text.

applying microwave (MW) irradiation, we can coherently manipulate the spin populations between these levels and prepare any qubit superposition state, whose spin dynamics can be represented using the Bloch sphere. All sensing methods demonstrated in this work, rely on the fundamental concepts explained in the following paragraphs.

1. *MW control of the V_B^- qubits*

The V_B^- spin-system can be described by the Hamiltonian $\hat{H} = \hat{H}_0 + \hat{H}_{\rm MW}$, where $\hat{H}_0$ represents the free evolution Hamiltonian under the effect of an external static magnetic field $\vec{B}_0 = B_0\hat{z}$ (Zeeman interaction) and $\hat{H}_{\rm MW}$ accounts for the MW control. In the following, the V_B^- center is treated as a pseudo-spin-1/2 system for simplicity. The Hamiltonian $\hat{H}_0$ in a laboratory frame of reference, where the V_B^- spin quantization axis is assumed to correspond to the z-axis (assuming $\hbar = 1$), reads:

$$\hat{H}_0 = \gamma B_0 \hat{S}_z = \omega_0 \hat{S}_z \,. \tag{1}$$

where γ is the electron gyromagnetic ratio, $\hat{S}_z$ the z-component of the spin operator $\vec{S}$, and ω_0 the V_B^- electronic spin precession frequency. The Hamiltonian $\hat{H}_{\rm MW}$ for the microwave interaction can be written as:

$$\hat{H}_{\rm MW} = 2\omega_1 \cos(\omega_{\rm MW} t + \phi_{\rm MW})\hat{S}_x \,, \tag{2}$$

where ω_1 is the MW amplitude, $\omega_{\rm MW}$ and $\phi_{\rm MW}$ are its frequency and phase, respectively. To drive the V_B^- qubit, the system is subjected to a MW field with a resonant frequency $\omega_{\rm MW} \approx \omega_0$, and with a MW polarization ($\hat{S}_x$) transverse to the quantization axis of the spin. We can simplify this Hamiltonian by transferring it to a rotating frame of reference, where it becomes:

$$\hat{\hat{H}} = \Delta\omega_0 \hat{S}_z + \omega_1 \hat{S}_x \,, \tag{3}$$

with $\Delta\omega_0 = \omega_{\rm MW} - \omega_0$. On resonance, $\Delta\omega_0 = 0$, the Hamiltonian describes the rotation of the spin vector around the x-axis with frequency ω_1. If we apply such a perturbation ($\hat{H}_{\rm MW}$) long enough for the spin to rotate by an angle of $90°$ from the starting position (z-axis), an equal superposition would be created, and the spin vector would lie on the $-y$ axis (Supplementary Figure 3 (a)).

2. *Interaction with external RF fields*

The V_B^- spin responds to the longitudinal (z) component of an RF field which is parallel to the V_B^- quantization axis and perpendicular to the flake's plane, which can be expressed as: $\vec{B}_{\rm RF}(t) = [b_{RF}\sin(2\pi\nu_{\rm RF} t + \phi_{\rm RF})]\hat{z}$. In our experimental setup, we optimized the position of our loop antenna such to generate oscillating magnetic fields preferentially along the z-axis. The Hamiltonian in the rotating frame takes the form:

$$\hat{\hat{H}} = \Delta\omega_0 \hat{S}_z + \hat{H}_{\rm RF} = \Delta\omega_0 \hat{S}_z + \gamma b_{\rm RF}\sin(2\pi\nu_{\rm RF} t + \phi_{\rm RF})\hat{S}_z \,. \tag{4}$$

Such a Hamiltonian causes the Bloch vector to rotate around the z-axis and accumulate a phase θ, that is the angle between the Bloch vector and its starting position (y-axis) (Supplementary Figure 3 (b)). However, due to the oscillating nature of the RF field, a periodical accumulation and cancellation of the phase θ will occur.

3. *Phase accumulation in a spin-echo experiment*

The detection of magnetic fields by V_B^- quantum sensors relies on the accumulation of a phase θ on the Bloch sphere. One example of this process is the spin-echo sensing method (schematically depicted in Supplementary Figure 4

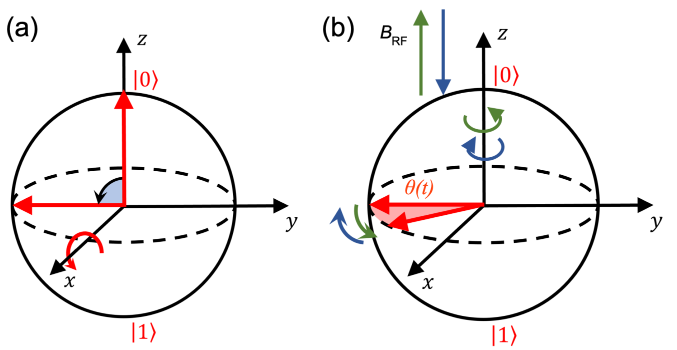

Supplementary Figure 3. (a) The spin-state superposition is prepared by a $\pi/2$ MW pulse. (b) The spin superposition responds to an oscillating magnetic field by accumulating a phase θ on the $x-y$ plane.

(a)), which is simple, yet forms the basis of more complex experiments presented in this work. The main function of the spin-echo is to refocus the V_B^- ensemble dephasing such that the limiting measurement time becomes the decoherence time T_2 rather than the much shorter dephasing time T_2*.

The spin dynamics occurring during the experiment is depicted in Supplementary Figure 4 (b)-(f). A laser pulse polarizes the V_B^- spins in the $|0\rangle$ state and a microwave $\pi/2$-pulse causes a 90° rotation of the Bloch vector around the x-axis. Assuming the first half-wave of the RF field points in the $+z$-direction, the Bloch vector starts rotating counterclockwise on the equatorial plane (green arrows) and accumulates a certain phase. Exactly at the time of zero crossing, when the magnetic field changes its sign, a π pulse (180° rotation around the x-axis) is applied, mirroring the position of the Bloch vector on the equatorial plane. The change of the sign of the magnetic field, now pointing in the $-z$-direction, causes an inversion of the rotational direction: The Bloch vector now moves clockwise (blue arrows), further away from the y-axis and thus accumulates more phase during the second half-wave of the magnetic field. Finally, the accumulated phase is mapped onto a population difference between the $|0\rangle$ and $|1\rangle$ states by a $\pi/2$-pulse, which is read out optically by a laser pulse. In order to sense an RF field of a specific frequency ν_{RF}, the spacing τ between the pulses is swept and the fluorescence signal is recorded. If the matching condition $\tau = \frac{1}{2\nu_{\mathrm{RF}}}$ is fulfilled, phase is accumulated and a dip in the fluorescence occurs (see Supplementary Figure 4 (g)). For frequencies not fulfilling the condition, a partial or total cancellation of phase would occur, hence the spin-echo acts as a spectral filter with a bandwidth which is approximately equal to $1/t_s$, where t_s is the duration of the pulse sequence. Considering a time-dependent magnetic field $B_{\mathrm{RF}}(t)$, since $\frac{d\theta}{dt} = \gamma B_{\mathrm{RF}}$, the total accumulated phase can be calculated by [4, 5]:

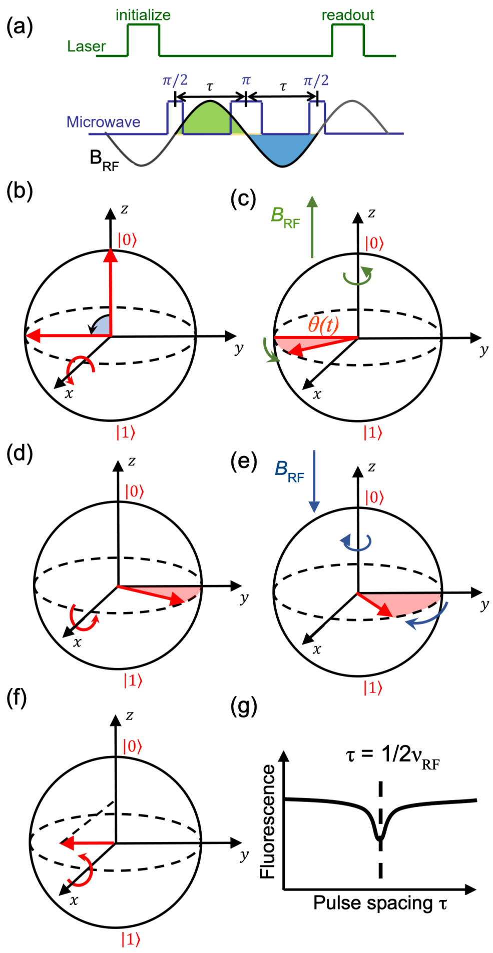

Supplementary Figure 4. (a) Spin-echo pulse sequence. (b) Time evolution of the Bloch vector in the spin-echo experiment. Initialization and $\pi/2$-pulse. (c) First free evolution time τ. (d) Control π-pulse. (e) Second free evolution time τ. (f) $\pi/2$ projection pulse and readout. (g) Sketch of the fluorescence dip at the matching condition $\tau = \frac{1}{2\nu_{\mathrm{RF}}}$.

$$\theta_{\mathrm{echo}} = \gamma \int_0^{\tau} b_{\mathrm{RF}} \sin(2\pi\nu_{\mathrm{RF}} t + \phi_{\mathrm{RF}}) dt \\ - \gamma \int_{\tau}^{2\tau} b_{\mathrm{RF}} \sin(2\pi\nu_{\mathrm{RF}} t + \phi_{\mathrm{RF}}) dt \,, \tag{5}$$

where the spin-flip by the π-pulse was taken into account by the sign change. The solution is:

$$\theta_{\mathrm{echo}} = \frac{4}{\pi} \gamma b_{\mathrm{RF}} \tau \cos(\phi_{\mathrm{RF}}) \,. \tag{6}$$

The phase accumulation is at its maximum when the signal phase ϕ_{RF} equals zero or an integer multiple of π. In this case, the phases of time intervals 1 and 2 in Supplementary Figure 5 (a) add up. On the other hand, if ϕ_{RF} is a half-integer

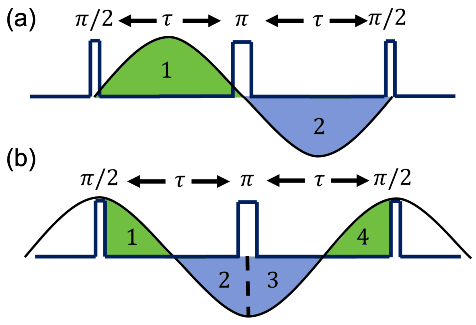

Supplementary Figure 5. Phase accumulation dependence on signal phase ϕ_{RF}. (a) Maximum phase accumulation (θ) (b) No phase accumulation (θ).

multiple of π, the overall accumulated phase is zero, as shown in Supplementary Figure 5 (b): Here, the accumulated phases during intervals 1 and 2 cancel each other out, as do the phases in the time intervals 3 and 4.

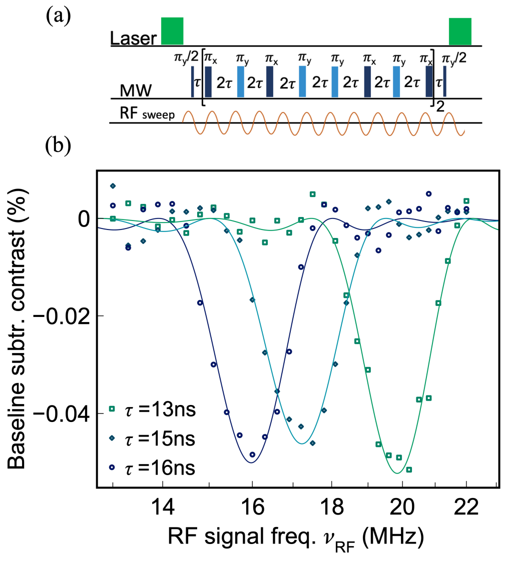

Supplementary Figure 6. (a) XY8-2 pulse sequence. (b) Probed filter functions using the pulse sequence in (a)

4. *Multipulse dynamical decoupling (pDD) schemes*

It is possible to extend the spin-echo pulse sequence by incorporating additional refocusing π-pulses, so that: 1) The decoherence time can be extended, as the width of the filter function is reduced and the V_B^- centers become less sensitive to magnetic noise beyond the bandwidth. 2) The extended decoherence time increases the time to interrogate the sensing RF field, meaning that more phase is accumulated after each π pulse, which increases the sensitivity of a measurement.

The maximum phase accumulation θ_{pDD} at the matching condition over an N π-pulse sequence is the same as for the spin-echo experiment (equation (6)), scaled by the number of pulses:

$$\theta_{\mathrm{pDD}} = N\theta_{\mathrm{echo}}\,. \tag{7}$$

In this work, pulsed dynamical decoupling (pDD) is achieved using the Carr-Purcell-Meiboom-Gill (CPMG) and the XY8-N pulse sequences. The first employs N control π-pulses that are spaced by 2τ (see Figure 2 (a) of the main text), and rotate the V_B^- spin about the same axis as it was prepared by the initial $\frac{\pi}{2}$ pulse. In contrast, the XY8-N sequence differs only in the pattern of MW-pulse phases which changes the rotation axis at each π-pulse in order to minimize the effect of pulse errors (see Supplementary Figure 6 (a)). In both cases, the filter function exhibits a typical sinc function shape:

$$F(\nu_{\mathrm{RF}}) \propto \frac{\sin(\pi\nu_{\mathrm{RF}}N2\tau)}{\pi\nu_{\mathrm{RF}}N2\tau}\,. \tag{8}$$

Here, the center frequency f_k is given by $f_k = \frac{k}{4\tau}$ where $k = 1, 3, 5, \ldots$ is the harmonic order. For a protocol of N pulses, with a total measurement time of $t_s = 2N\tau$, the width of the filter Δf is given by $\Delta f = 1/t_s$. For large N, the filter function becomes peaked and the bandwidth narrow.

In order to experimentally probe the filter function, which essentially dictates the sensing behaviour of the V_B^- spin defect for a given multipulse sequence, the microwave pulse spacing was held constant at a specific matching condition while ν_{RF} was swept. Supplementary Figure 6 shows the spectral response of the V_B^- sensor as fluorescence contrast dips at three different matching conditions $\tau = 1/(4\nu_{\mathrm{RF}})$ for an XY8-2 sequence, illustrating the characteristic bandpass behaviour. For the experiments reported here, $t_s \approx 500$ ns and the bandwidth $\Delta \approx 2$ MHz, as expected.

5. *Slope and variance detection*

The behavior of the V_B^- sensor in response to changes in magnetic field strength (b_{RF}) is dependent on how the pulse sequence has been configured to measure the final spin state (see visual explanation in Supplementary Figure 7). This dependence can be calculated through the expectation value of the spin's z-component that can be directly translated to fluorescence intensity. This will give us:

$$\langle S_z \rangle = \frac{1}{2}\cos(\theta - \phi_{\mathrm{MW}})\,, \tag{9}$$

where ϕ_{MW} is the relative microwave phase of the last $\frac{\pi}{2}$ projection pulse. For $\phi_{\mathrm{MW}} = \frac{\pi}{2}$, the rotation induced by the last $\pi/2$-pulse is around the y-axis, yielding: $\langle S_z \rangle = \frac{1}{2}\sin\theta$ (Supplementary Figure 7 (b)). Whereas, for $\phi_{\mathrm{MW}} = 0$, the last $\frac{\pi}{2}$ rotation is around the x-axis, which gives us:$\langle S_z \rangle = \frac{1}{2}\cos\theta$ (Supplementary Figure 7 (c)). Such a configuration sets the bias point of the measurement, that is, a reference measurement signal (such as fluorescence intensity) obtained in the absence of an RF field. Thus, depending on where

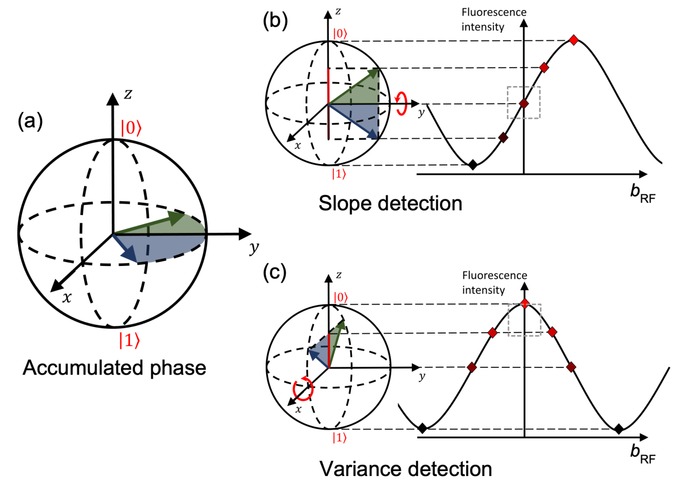

Supplementary Figure 7. Slope and variance detection. (a) Positive and negative accumulated phases as indicated by the green and blue arrows. (b) Signal-phase sensitive slope detection mode is set by a $(\frac{\pi}{2})_y$ projection pulse. (c) Signal-phase insensitive variance detection mode is achieved by applying a $(\frac{\pi}{2})_x$ projection pulse.

the bias point is positioned, the magnetometry mode can be characterized by: i) slope detection where, for small signal changes, the sensor's response is linear. ii) variance detection, where small signal changes lead to a quadratic sensor's response (see grey dotted boxes in Supplementary Figure 7 (b) (c)). The slope detection mode is best implemented in the case of coherent signals whose phase can be locked in regards to the pulse sequence. This is for instance the case for free induction decays (FID) arising from Boltzmann distributions of spins in microscale samples. However, for stochastic signals such as spin noise, the accumulated phase can occur in both directions and averaging in slope detection mode will lead to a cancellation of the detected signal. On the other hand, in the case of the phase-insensitive variance detection mode, both directions of the accumulated phase give the same quadratic fluorescence response. Therefore, this variance detection scheme is well-suited for measuring noise signals with random amplitudes and phases. This is for instance the case for randomly fluctuating NMR signals (spin noise) originating from nanoscale samples. To demonstrate the possibility of using these methods both for nanoscale and microscale spin sensing, the DD protocols in the section "Sensing RF Signals with V_B^- defects in hBN" (XY8-N and spinlock sequences) were operated in variance detection mode, whereas the CASR protocol in the section "Sensing of RF signals with arbitrary frequency resolution" employs phase-sensitive XY8-2 schemes working in slope detection mode.

6. *Experimentally probed phase accumulation with V_B^- in hBN*

Finally, we probe the RF phase and amplitude dependence. We set the XY8-1 and XY8-2 protocols to the phase-sensitive slope detection mode and monitor the V_B^- fluorescence contrast while sweeping through different RF phases using a constant RF amplitude (Supplementary Figure 8 (a), (c), (e)) and different RF amplitudes using a constant RF phase (Supplementary Figure 8 (b), (d), (f)). By combining equations (8) and (9), it is possible to fit the experimental data, proving that sensing RF magnetic fields with V_B^- centers in hBN follows the same relations for phase accumulation expected in theory.

For the phase-sweep experiments, we keep the RF amplitude b_{RF} constant by setting the signal source output voltage (peak-to-peak voltage) at a value of 0.2 V_{pp}. Thus, we fit the data with the following equation:

$$\begin{aligned} &A\cos(\theta-\phi_{\mathrm{MW}})-B= \\ &=A\cos\left(\frac{4}{\pi}\gamma b_{\mathrm{RF}}N\tau\cos\left(\frac{\phi_{\mathrm{RF}}+\Delta\phi_{\mathrm{RF}}}{180}\pi\right)-\frac{\pi}{2}\right)-B\,. \end{aligned} \tag{10}$$

The x-variable is in this case the swept RF phase ϕ_{RF} and the constant parameters are the gyromagnetic ratio $\gamma = 1.76\times10^{11}$ rad s^{-1}T^{-1}, $N = 8, 16$ is the number of π-pulses for the XY8-1 and XY8-2 sequences, respectively, τ the interpulse delay for matching an RF frequency of 16 MHz, $\tau = 1/(4\nu_{\mathrm{RF}}) = 15.6\,\mathrm{ns}$. The data are shown in Supplementary Figure 8 (c), (e) and the fitting parameters are summarized in Supplementary Table 1:

Supplementary Table 1. Fitting parameters for the phase sweep experiment

Prot.	A (a.u.)	B (a.u.)	b_{RF} (μT)	$\Delta\phi_{\mathrm{RF}}$ (°)
XY8-1	(28±1)×10^{-4}	(-1.3±0.4)×10^{-4}	53±4	49.2±1.1
XY8-2	(6.9±0.2)×10^{-4}	(-2.0±0.1)×10^{-4}	52±1	48.4±0.9

A and B are normalization and offset terms, b_{RF} is the strength of the RF field, $\Delta\phi_{\mathrm{RF}}$ is a phase offset accounting for the actual phase relation between the RF signal and the pulse sequence which is set by instrumental timings.

The amplitude-sweep experiments involve maintaining the phase at an optimized value of 315° while sweeping the RF amplitude b_{RF} by varying the output voltage of the signal source (in V_{pp}). The resulting fluorescence contrast is fitted using the same relation as before. However, in this case, the x-variable is the amplitude b_{RF} and is multiplied by κ, a conversion factor (T/V_{pp}) that we estimate by fitting the data and enables us to determine the actual magnetic field (in Tesla) sensed by the sensor. The data are shown in Supplementary Figure 8 (d), (f) and the fitting parameters are summarized in Supplementary Table 2:

Supplementary Table 2. Fitting parameters for the amplitude sweep experiment

Prot.	A (a.u.)	B (a.u.)	κ (T/V_{pp})
XY8-1	(3.2±0.1)×10^{-3}	(1.2±1.0)×10^{-4}	(2.24±0.02)×10^{-4}
XY8-2	(7.8±0.4)×10^{-4}	(1.8±0.3)×10^{-4}	(2.23±0.03)×10^{-4}

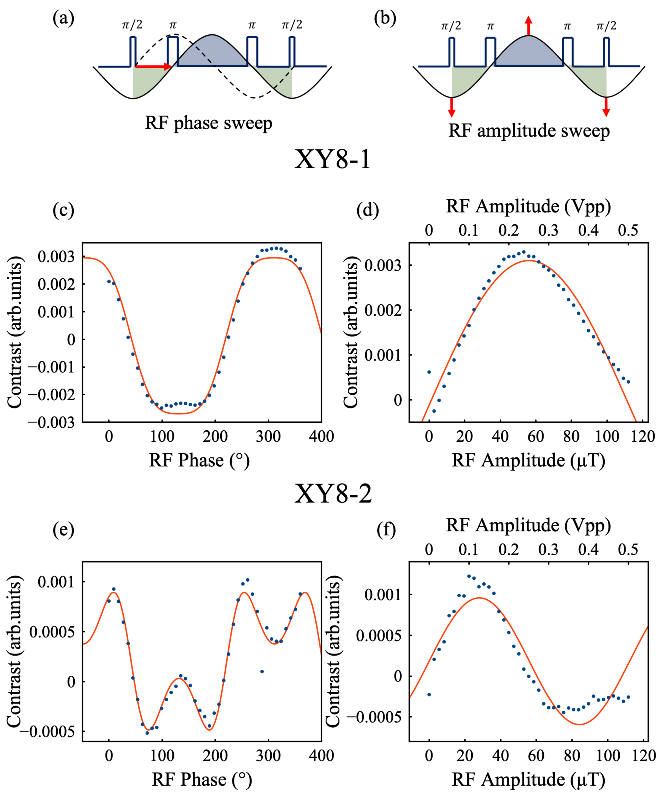

Supplementary Figure 8. XY8-N signal phase and amplitude sweep. For the XY8-1 and XY8-2, the RF phase is swept ((a), (c) and (e)) in order to identify the signal phase of maximum phase accumulation on the Bloch sphere. This phase is chosen for the amplitude calibration experiment ((b),(d) and (f)), whereby the RF amplitude is swept. From this, the accumulated phase in dependence of the magnetic field amplitude can be directly observed.

7. *Phase accumulation in the spinlock experiment*

The rotating-frame Hamiltonian $\hat{H}_{\text{SL,rot1}}$, accounting for a spinlock pulse of amplitude Ω around the y-axis and an RF signal $B_{\text{RF}}(t) = b_{\text{RF}}\cos(2\pi\nu_{\text{RF}}t + \phi_{\text{RF}})$ along the z-axis, reads (in natural units $\hbar = 1$):

$$\hat{H}_{\text{SL,rot1}} = \Omega\,\hat{S}_y + \gamma b_{\text{RF}}\cos(2\pi\nu_{\text{RF}}t + \phi_{\text{RF}})\,\hat{S}_z\,, \qquad (11)$$

with the spin operators $\hat{S}_y$ and $\hat{S}_z$. This equation can be transferred into a second rotating frame of the Rabi oscillation, where the first term vanishes. Assuming that the matching condition of $\Omega = 2\pi\nu_{\text{RF}}$ is fulfilled and that the rotating wave approximation for $\Omega \gg \gamma b_{\text{RF}}$ is valid, the Hamiltonian $\hat{H}_{\text{SL,rot2}}$ in the second rotating frame becomes:

$$\hat{H}_{\text{SL,rot2}} = \frac{1}{2}\gamma b_{\text{RF}}\left(\cos(\phi_{\text{RF}})\hat{S}_z + \sin(\phi_{\text{RF}})\hat{S}_x\right)\,. \qquad (12)$$

Setting the signal phase to $\phi_{\text{RF}} = 0$ results in a precession of the Bloch vector around the z-axis on the equatorial plane with an angular velocity of $\frac{d\theta}{dt} = \frac{1}{2}\gamma b_{\text{RF}}$. Thus, the accumulated phase θ_{SL} during the spinlock pulse is

$$\theta_{\text{SL}}(t) = \frac{1}{2}\gamma b_{\text{RF}}t\,. \qquad (13)$$

As mentioned in the previous sections, the dynamical sensing behaviour of the V_B^- defect is determined by the filter function, centered around the matching condition, $\Omega = 2\pi\nu_{\text{RF}}$, allowing for an accumulation of phase within the filter bandwidth. In order to probe the shape of the filter function experimentally, the RF signal frequency ν_{RF} was swept for different (fixed) spinlock amplitudes $\Omega = 2\pi\nu_R$, as shown in Supplementary Figure 9. Here, the labels next to each dip indicate different values of ν_R normalized to a maximum spinlock amplitude corresponding to $\nu_R^{(1)} \approx 18\,\text{MHz}$. As expected, the fluorescence dip shifts to higher frequencies for an increasing spinlock amplitude. Furthermore, the dip's intensity also increases accordingly, since the spinlock becomes more and more efficient at locking the spins (see Raw data in Supplementary Note 9). Recalling that the spinlock's phase accumulation mechanism relies on promoting dressed state spin transitions by the sample RF field, the sensor's bandwidth Δf, is set by the parameters limiting this process, such as primarily the duration and strength of the driving field. Thus, if the RF is continuously irradiating the system, the spinlock time t_{SL}, limits the filter function as: $\Delta f = 5.5680 t_{\text{SL}}^{-1}$, if $t_{\text{SL}}^{-1} > \gamma b_{\text{RF}}$, and the RF field strength: $\Delta f = 2\gamma b_{\text{RF}}$, if $t_{\text{SL}}^{-1} < \gamma b_{\text{RF}}$ [6]. In addition, the inhomogeneous broadening of the electron spin resonance (ESR) spectrum and the MW field inhomogeneities throughout the V_B^- ensemble may result in a range of effective Rabi frequencies that can significantly impact the sensor's bandwidth [6, 7]. Compared to the measured filter functions using the XY8-2 protocol (Supplementary Figure 6), the filter functions in the spinlock experiments exhibit broader bandwidth ($\Delta f \approx$4-6 MHz). However, this broadening is attributable uniquely to the short spinlock time used in the experiments, since $t_{\text{SL}} = 0.5\,\mu\text{s}$. This suggests that even though the V_B^- ESR spectrum is broad, the excited off-resonant spin packets have little impact on the spinlock process, most likely due to fast dephasing of these components, which are not "locked" by the MW pulse. This results in a limited contribution from these spin packets to the overall dip's lineshape, in contrast with what observed in the case of ensemble of NV-centers in diamond where dephasing times are significantly longer [7].

8. *Coherently Averaged Syncronized readout (CASR) and estimation of sensitivity*

In the section of the main text "Sensing of RF signals with arbitrary frequency resolution", the CASR method [8–10] is demonstrated for V_B^- defects in hBN. The basic concept of this method is to synchronize a train of pulsed dynamical decoupling sequences (such as XY8-N) with RF magnetic field to be captured. Each pDD sequence (including laser initialization and readout) is operated in the phase-sensitive slope detection mode (for linear detection, see Supplementary Note 3) and is tuned to the sample RF frequency by satisfying the condition $\tau = 1/(4\nu_{\text{RF}})$, such that: $\nu_{\text{pDD}} = \nu_{\text{RF}} = 1/(4\tau)$. The pDD sequence is then repeated multiple times, such that the time between successive sequences is an integer

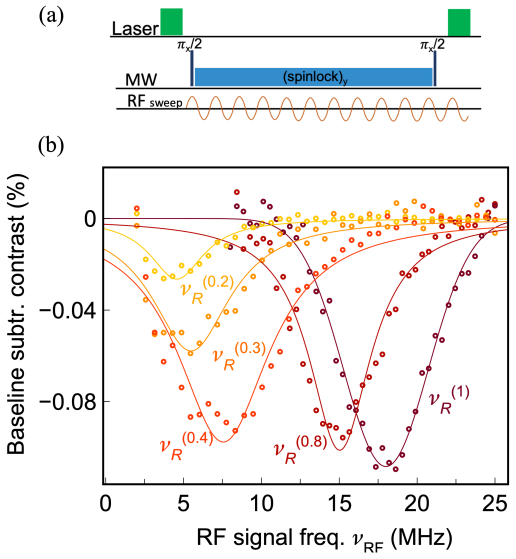

Supplementary Figure 9. (a) Spinlock pulse sequence. (b) Probed filter functions using the pulse sequence in (a).

multiple of the B_{RF} period T_{RF}. In this way, every dynamical decoupling sequence experiences an identical phase of the RF field, resulting in the same fluorescence readout for each individual step. However, if the frequency ν_{RF} deviates slightly from ν_{pDD}, the magnetic field advances in phase for each pDD readout, causing a downconverted fluorescence contrast signal that oscillates at the difference frequency between the pulse sequence (ν_{pDD}) and ν_{RF}, given by $\Delta\nu = \nu_{\mathrm{pDD}} - \nu_{\mathrm{RF}}$. Running the protocol for a measurement time on the order of seconds, allows us to acquire a time-trace that can be Fourier transformed to yield signals with sub-Hertz linewidth.

In the experiment described in the main paper, a train of XY8-2 sub-sequences is synchronized with the RF signal at a detuning frequency of $\Delta\nu = 1000\,\mathrm{Hz}$. In addition to these measurements, we employ the CASR method to investigate the sensitivity of our setup experimentally. For the following measurements, the laser spot diameter was set to $10\,\mu\mathrm{m}$. In the experiments presented in Supplementary Figure 10, we sweep the amplitude of the external RF field b_{RF} and, for each amplitude value, we conduct a CASR experiment and capture the corresponding time trace (Supplementary Figure 10(a)). Then, we extract the information about the V_B^- phase accumulation at each RF field amplitude by monitoring the amplitude of the CASR oscillating time-domain signal at a single time point (for instance, at an anti-node, as shown by the red dots in Supplementary Figure 10 (a)). Thus, the chosen anti-node amplitudes are plotted as a function of the applied voltage of the RF signal source (see Supplementary Figure 10 (b)). The magnetic field per applied voltage is calculated as described in Supplementary Note 3. In this case, the voltage generates an RF amplitude $b_{\mathrm{RF}}/V_{\mathrm{pp}} \approx 375\ \mu\mathrm{T}/V_{\mathrm{pp}}$. Thus, to obtain the estimation of sensitivity, we performed a CASR experiment setting our RF output voltage to 0.05 V_{pp} ($b_{\mathrm{RF}} = 17.5\ \mu\mathrm{T}$) and acquiring the signal for 1 second without averaging (Supplementary Figure 10 (c)). We estimated the $\mathrm{SNR} = A_{peak}/\sigma_{noise} \approx 8.5$, where A_{peak} is the signal's peak amplitude, and σ_{noise} is the standard deviation of the complex noise spectrum. This allows us to calculate the sensitivity as: $\eta = b_{\mathrm{RF}}/\mathrm{SNR} \approx 2\ \mu\mathrm{T\ Hz}^{-1/2}$.

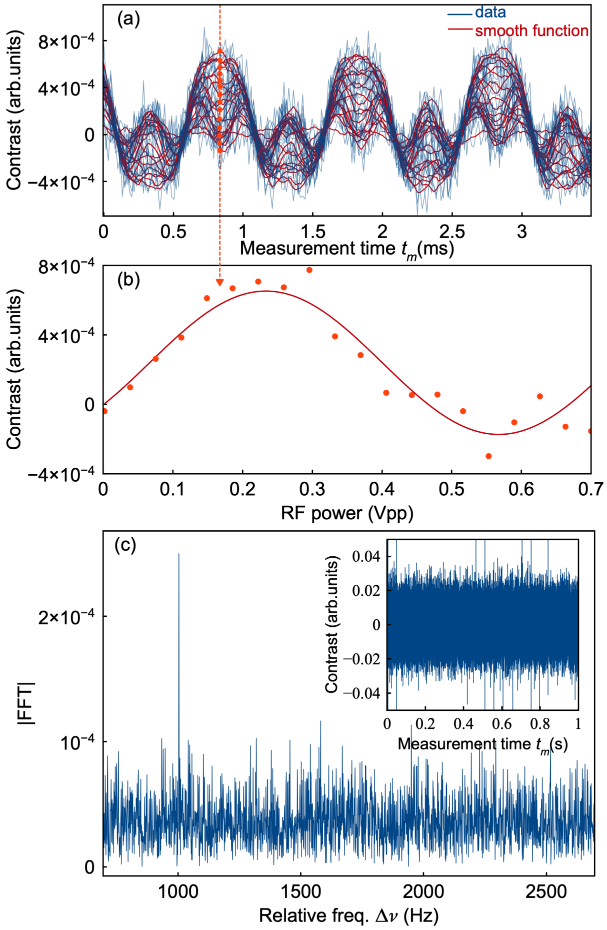

Supplementary Figure 10. (a) CASR Time traces in the first 3.5 ms, showing how the signal oscillation's amplitudes vary with varying RF field amplitude b_{RF}. A smooth function was used as an aid for data visualization. (b) One of the anti-nodes is chosen, indicated by the red circles in (a) and the signal amplitude at this time-point is plotted as a function of the applied Voltage of the RF signal source. (c) CASR spectrum obtained upon |FFT| of the time trace in the inset. The experiment was performed in one single shot for a total measurement time of 1 second.

Supplementary Note 4: Extension of the coherence times by the XY8-N protocol

We utilized the XY8-N pulse sequence to maintain the coherence of V_B^-, as demonstrated in the experiments presented in Figure 2(a) of the main text. The decoherence curves were fitted with a stretched exponential: $a \cdot (\exp(t_s/T_2)^c)$ with $c \sim 1$ and $t_s = 2N\tau$. The fitted coherence time values are plotted versus the number of pulses (see Supplementary Figure 11, inset), and show a sub-linear dependence $f(N) \propto N^s$ where $s \sim 0.52$. With XY8-N, we observe a drop in contrast already after $\sim$ 100 pulses (vs. 1000 with CPMG) which sets our limit for observing a further extension of the coherence time. This allows us to probe a maximal extension by a factor $\sim$ 6 with respect to the spin-echo T_2 time. As is clearly visible, the XY8-N sequence still extends the V_B^-coherence time, however showing overall worse performance in preserving the prepared spin state (S_x) with respect to the CPMG experiment. On the other hand, when sensing RF fields applied under the same conditions as in the XY8-2 experiments, a CPMG sequence with 16 pulses yielded no appreciable signal. This result indicates that the XY8-N sequence is more sensitive than the CPMG in detecting RF fields. The reason for this disparity is likely due to the nature of the CPMG technique, which is a pulsed spinlock protocol designed to effectively preserve the spin state along the axis in which the initial state was prepared. However, it has the consequence of eliminating all components that are perpendicular to this axis. In contrast, the XY-protocol is less efficient at "locking" the spin state onto a specific axis, but it compensates for pulse errors and better preserves an arbitrary spin state that may have developed perpendicular components due to phase accumulation [11–13].

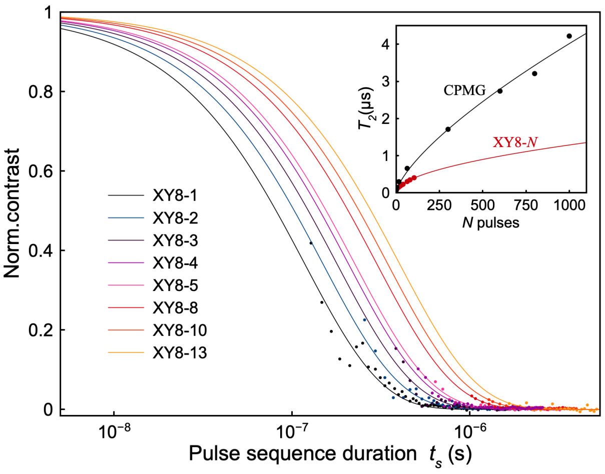

Supplementary Figure 11. Extension of the coherence times by using XY8-N protocols. The experiments have been conducted as in the Figure 2 (a) of the main text, monitoring the fluorescence contrast while sweeping the inter pulse delay τ. The resulting decoherence curves have been fitted with a monoexponential function. The modulation superimposed was neglected in this case. Inset: comparison of the CPMG and XY8-N protocols.

Supplementary Note 5: Estimation of sensitivity and comparison with NV-diamond sensors

We note that comparing two distinct systems, such as NV centers in diamond and V_B^- centers in hBN, involves some degree of approximation. Nevertheless, we will attempt to estimate the sensitivity of V_B^- sensors for CPMG-like protocols based on the theory presented in prior studies on NV centers in diamond [14, 15]. For slope detection, the sensitivity η, in units of $\mathrm{T\,Hz^{-1/2}}$, can be calculated through the expression:

$$\eta = \frac{\pi}{2}\frac{\hbar}{\gamma}\left(\frac{1}{\sqrt{n t_s}}\right)\left(\frac{1}{e^{-(\frac{N^{-s} t_s}{T_2})^c}}\right) \times \times \sqrt{1+\frac{1}{C^2 n_{avg}}}\sqrt{1+\frac{t_I+t_s+t_R}{t_s}} \tag{14}$$

Here, n represents the number of active spin defects. We estimated this value based on what was reported in [16], where a concentration of V_B^- defects on the order of $\sim$200 ppm was estimated for a similar sample (100 nm hBN thickness, He^+ implantation with 3 keV energy and a dose of approx. 1 ion nm^{-2}). Assuming a laser spot radius of approximately 5 μm (as is the case for the previously described CASR experiments) and a defect layer thickness of around 60 nm, as reported in [16], we estimate that the volume containing the simultaneously interrogated spin defects is approximately 5 μm^3, with a total of approximately $5{\times}10^7$ defects. The equation also includes several other variables. N represents the number of π-pulses, $t_s = \frac{N}{2\nu_{\mathrm{RF}}}$ is the duration of the spin interrogation time, and ν_{RF} is the frequency of the RF sensing field. The variable s is the exponent in the power law that describes the increase in T_2 by increasing the number of π-pulses. In our experiments, we measured $s_{\mathrm{CPMG}} \sim 0.71$ using the CPMG protocol (Figure 2(a)) and $s_{\mathrm{XY8}-N} \sim 0.52$ using the XY8-N protocol (Supplementary Figure 11 in Supplementary Note 4). The variable c represents the stretched exponent used to fit the coherence curves and depends on the specific dephasing mechanisms. In all cases, we found $c \sim 1$. C represents the fluorescence contrast, which is set to 0.05, and n_{avg} denotes the average number of collected photons per defect per measurement. We estimated the value of n_{avg} for V_B^- centers based on the values reported for NV centers in diamond, which provide $1{\times}10^5$ counts/s per NV [15]. The NV centers in diamond have a quantum efficiency (QE) of approximately 70 % [17], while V_B^- centers have a much lower QE of approximately 0.03 % [18]. Assuming similar collection efficiencies and absorbance properties, this results in a much lower fluorescence rate of 42 counts per second per V_B^- center. For a readout time of 300 ns, this leads to an average of n_{avg} = 42 counts per second $\times$ 300 ns = $1.26{\times}10^{-5}$ photons collected per V_B^- center per measurement, which is much lower than the $1{\times}10^5$ counts per second $\times$ 300 ns = $3{\times}10^{-2}$ photons collected for NV centers. Finally, the last term takes into account the optical spin-state initialization and readout times, t_I and t_R respectively, which cause the relevant spin-interrogation time

t_s to be only a fraction of the total measurement time. In our case, these parameters have been both set to the values: $t_I = 5\,\mu$s and $t_R = 300$ ns.

Using the approach described above, we compare the sensitivities of different solid-state spin systems, such as our V_B^- ensemble in the hBN sample, the shallow NV-ensemble in diamond used in Liu et al.[19], and the bulk NV-diamond in [15]. As shown in Supplementary Figure 12, we find that the V_B^- sensitivity is on the order of a few μT/$\sqrt{\text{Hz}}$, while the sensitivity of NV sensors is in the range of nT/$\sqrt{\text{Hz}}$. We note that these results are consistent with what is shown in Supplementary Note 3 where the V_B^- sensitivity was measured through a RF signal calibration procedure based on the CASR method.

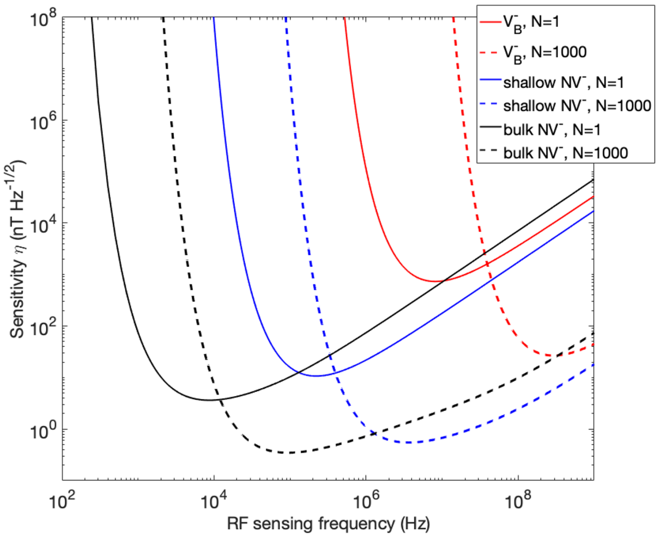

Supplementary Figure 12. Calculated dependence of the sensitivity η for ensembles of V_B^- in hBN (red line), near-surface NV-centers (as in Ref. [19], blue line) and bulk NV-centers in diamond (as in Ref. [15], black line). Each curve has been calculated based on equation (14). The parameters used in the calculation are summarized in Supplementary Table 3

.

Supplementary Table 3. Parameters for ensembles of V_B^- in hBN (this work), shallow NV centers (system used in Ref. [19]), and bulk NV centers in diamond (as in Ref. [15]).

Sample	T_2 (μs)	n	c	s	C	n_{avg}
hBN	0.06	4.7×10^7	1	0.5	0.05	1.3×10^{-5}
Near-surface NV	4	7.5×10^4	1	0.5	0.05	0.03
Bulk NV	160	4.5×10^3	1	0.5	0.05	0.03

It is important to consider that the sensitivities mentioned above are specifically estimated for an RF signal that is synchronized with the detection sequence (slope detection, see Section 5 of Supplementary Note 3). In a simplified explanation, this is dependent on the spin coherence times (T_2), as described by the relationship: $\eta_{\text{slo}} \propto \frac{1}{\sqrt{T_2}}$ [14, 15, 20]. However, when detecting non-coherent RF fields, the sensitivity exhibits a stronger dependence on T_2, as illustrated by: $\eta_{\text{var}} \propto \frac{1}{\sqrt{T_2^3}}$[20, 21]. This determines a short T_2 time to have a dramatic impact on sensitivity. Thus, particularly for applications that rely on spin-noise detection, such as nanoscale NMR sensing, the extension of the T_2 time is of paramount importance to achieve notable sensor performance. Another possibility to overcome these challenges is to utilize spin hyperpolarization techniques [22, 23]. These techniques offer a solution by enabling detection of nuclear coherent signals even at these small scales.

Supplementary Note 6: Optimizing conditions for sensing RF fields with V_B^- in hBN

The sensitivity of the RF sensing protocols based on spin-echo schemes is limited by the T_2 time, with the optimal spin interrogation time being on the order of $t_s \sim T_2$ [14, 15]. For CPMG-like sequences, the sensitivity can be further enhanced by using N π-pulses that extend the T_2 time. However, the number of π-pulses used increases the measurement time linearly, whereas the T_2 time increases sub-linearly. As a result, for a given RF frequency to sense, an optimal number of pulses can be identified. Using the approach described above we estimate the optimal pulse sequence setup for detecting RF in a certain frequency range. The resulting plots show the sensitivity dependence on the RF sensing frequency and provide insight into the optimal number of pulses for each frequency. As depicted in Supplementary Figure 13 (a), a spin-echo ($N = 1$) sequence gives the best performance for frequencies up to ~7-8 MHz (blue shaded area), whereas for ν_{RF} =10 MHz frequency, the best predicted sequence is with N=8 π-pulses (red line and shaded area). At $10 < \nu_{\text{RF}} < 30$ MHz, the four sequences exhibit similar performance as predicted, whereas for the highest achieved sensing frequency of 42 MHz, the optimal sequences involve 16-32 π-pulses (green shaded area). These results are confirmed qualitatively through experiments (spin-echo, XY8-1,2,3, and 4) for $\nu_{\text{RF}} =$ 6, 10, 25, and 40 MHz sensing frequencies (see inset in Supplementary Figure 13 (a)). We observe that the XY8-4 sequence does not exhibit better performance than the XY8-3 sequence for $\nu_{\text{RF}} \approx 40$ MHz, as expected. This may be due to the accumulation of pulse errors, which were not accounted for in the simulation.

The optimal number of pulses for a given sensing frequency can also be estimated with the following relation [14, 15]:

$$N_{opt} = \left(\frac{1}{2c(1-s)} (2T_2\nu_{\text{RF}})^c \right)^{\frac{1}{c(1-s)}} . \qquad (15)$$

The result is plotted in Supplementary Figure 13 (b).

In general, XY8-N sequences with 8-24 π-pulses (XY8-1 to XY8-3) allowed for an easy detection of RF signals in the 10-40 MHz range. In particular, XY8-1 resulted ideal to sense frequencies in the 8-15 MHz range, whereas XY8-2 for 15-30 MHz and XY8-3 for frequencies above 30 MHz. At the low end, the XY8-N sequence is limited by the coherence time, making it challenging to measure frequencies below 6-7 MHz

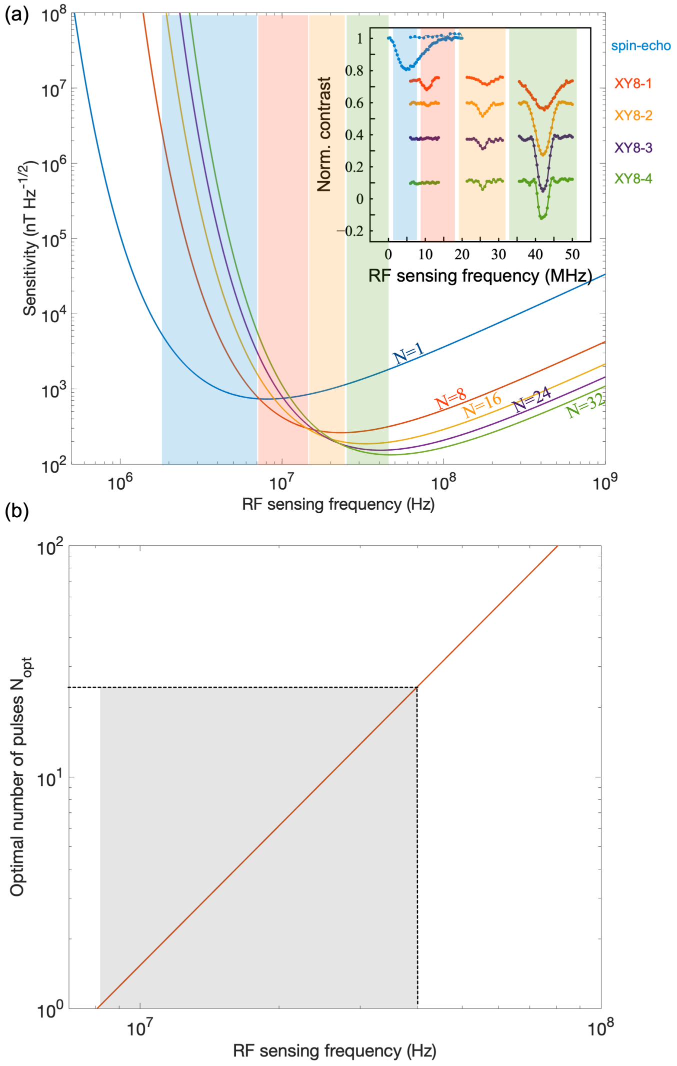

Supplementary Figure 13. (a) Calculated dependence of the sensitivity η of multipulse dynamical decoupling sequences on the RF sensing frequency. Each curve has been calculated based on equation (14) using $T_2 = 62$ ns, $c = 1$, $C = 0.05$, $s = 0.5$ and $N = 1, 8, 16, 24, 32$ (see labels). Color coded is the correspondence with spin-echo, XY8-1,2,3,4 experiments in the inset. Inset: the dips have been recorded keeping τ fixed while sweeping the RF frequency. The values of τ were chosen to match conditions for 6, 10, 25 and 40 MHz sensing and all spectra were recorded with the same experimental conditions. Color shaded areas indicate the comparison between the frequency range in the calculated plot and the corresponding experimental spectra in the inset. (b) Plot of equation (15) showing the dependence of the optimal number of pulses on the RF field frequency. The shaded grey area represents the range of conditions that were utilized in our experiments.

due to a required inter-pulse spacing of 100 ns (T_2(XY8-1) $\approx$ 120 ns, T_2(XY8-2) $\approx$ 150 ns). At the high end, we were able to measure a maximum frequency of more than 40 MHz, the limit being set by the maximum microwave (MW) power, which determines the minimal MW pulse duration.

Supplementary Note 7: Coherent manipulation of the V_B^- dressed-states

In the following experiments the evolution of the V_B^- dressed-states during matched spinlock is probed. Here, a spinlock pulse sequence is applied where the spinlock duration is gradually increased. The RF frequency is kept at a fixed value, matching the condition $\nu_{\mathrm{RF}} = \Omega/2\pi = 18$ MHz. The RF phase ϕ_{RF} is kept constant with respect to the pulse sequence. Furthermore, the last $\pi/2$-pulse has been removed (see Supplementary Figure 14 (a)) to directly detect the rotating-frame Rabi evolution as oscillations of the spin vector's z-projection. The observed signal is modulated at a frequency of 18 MHz, in accordance to the matching condition. Moreover, the phase of these oscillations is sensitive to the RF phase ϕ_{RF} utilized for driving the dressed-states (see Supplementary Figure 14 (b)). To, at least qualitatively, describe the dynamics occurring during the experiment, numerical simulations have been performed based on the density matrix evolution of a simple spin-1/2 system under the Hamiltonian reported in the equation (11) of Supplementary Note 3. The resulting density matrix is then corrected by an exponential term accounting for dressed-state decoherence using a bi-exponential decay with time constants of $T_a = 1.6 \times 10^{-7}$ s and $T_b = 1.5 \times 10^{-6}$ s with amplitudes of 0.95 and 0.05, respectively. These parameters have been chosen until the oscillation damping in the simulation resembled the experimental one. The simulation for $\phi_{\mathrm{RF}} = 180°$ with no relaxation correction is shown in (Supplementary Figure 14 (d)) for a comparison. We note that, due to the RF field amplitude not being strong enough to counteract the fast decoherence, the detected evolution is likely to be only the very initial part of the complete Rabi rotation from the $|+\rangle$ to $|-\rangle$ dressed-states (Supplementary Figure 14 (d)).

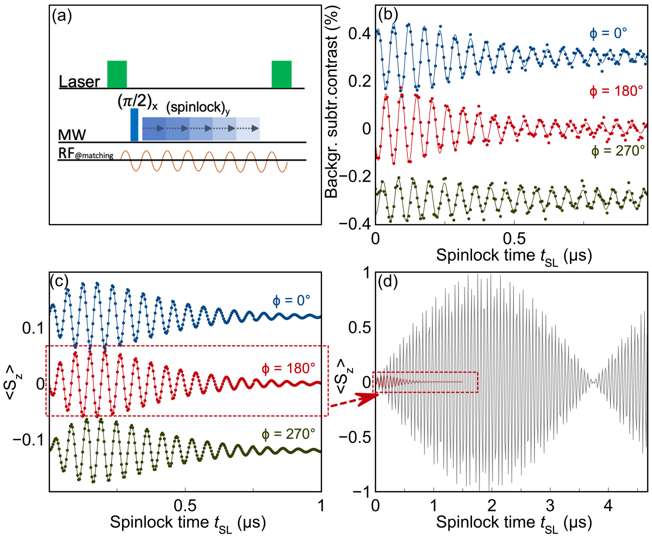

Supplementary Figure 14. (a) Spinlock pulse sequence. Each datapoint corresponds to the fluorescence contrast detected immediately after the spinlock pulse. (b) Experiments performed setting different ϕ_{RF} values resulting in different Rabi phases. (c) Numerical simulations of dressed-state Rabi nutation. (d) Overlap of the simulation reported in (c) (red line) and the same numerical simulation with $t_{\mathrm{SL}} \sim 5\,\mu\mathrm{s}$ in the absence of decoherence (grey).

Supplementary Note 8: Fitting Details

1. ODMR spectrum

Supplementary Table 4. Fitting parameters of the ODMR spectrum shown in Figure 1 (e).

HF Line	a(a.u.)	f_0(GHz)
1	(-0.58±0.02)×10^{-3}	3.075 ±0.001
2	(-1.21±0.02)×10^{-3}	3.120 ±0.001
3	(-2.50±0.03)×10^{-3}	3.164 ±0.001
4	(-2.50±0.03)×10^{-3}	3.208 ±0.001
5	(-2.50±0.03)×10^{-3}	3.249 ±0.001
6	(-1.00±0.03)×10^{-3}	3.293 ±0.001
7	(-0.47±0.03)×10^{-3}	3.333 ±0.002

2. T_2 measurement

Supplementary Table 5. Fitting parameters of the T_2 measurement in Figure 1 (h).

a	T_2(ns)	c	b	T_f(ns)	f(MHz)
0.94 ± 0.01	58.5 ± 0.4	1.03 ± 0.01	0.084 ± 0.007	71 ± 5	44.5 ± 0.1

3. Extension of the coherence times

Supplementary Table 6. Fitted coherence time constants and corresponding stretch-exponents c for the data shown in Figure 2 (a). Parameters with * have been kept locked in fitting.

N (π-pulses)	T_2 [ns]	c
1	63 ± 1	1.04 ± 0.03
4	138 ± 3	0.98 ± 0.04
16	294 ± 9	0.88 ± 0.04
64	653 ± 13	0.97*
300	1710 ± 370	1.12 ± 0.17
600	2730 ± 249	1.02 ± 0.06
800	3209 ± 265	1.00 ± 0.05
1000	4220 ± 493	1.06 ± 0.01

4. RF sensing with the XY8-2 pulse sequence

Supplementary Table 7. Fitting parameters for the data shown in Figure 3 (b).

ν_{RF} (MHz)	Expected τ (s)	Fitted τ (s)	Fitted N pulses
16	1.5625×10^{-8}	$(1.5586 \pm 0.0081)\times10^{-8}$	15.1 ± 1.4
18	1.3888×10^{-8}	$(1.4101 \pm 0.0045)\times10^{-8}$	15.4 ± 0.9

Supplementary Table 8. Fitting parameters for the data shown in Figure 3 (c).

	Expected τ (s)	Fitted τ (s)	Fitted N pulses
XY8-1	1.5625×10^{-8}	$(1.5854 \pm 0.0080)\times10^{-8}$	7.2 ± 0.3
XY8-2	1.5625×10^{-8}	$(1.5583 \pm 0.0081)\times10^{-8}$	15.1 ± 1.4
XY8-3	1.5625×10^{-8}	$(1.5518 \pm 0.0249)\times10^{-8}$	14.8 ± 4.1
XY8-4	1.5625×10^{-8}	$(1.5025 \pm 0.0910)\times10^{-8}$	6.6 ± 3.1

5. RF sensing with the spinlock pulse sequence

Supplementary Table 9. Fitting parameters for the data shown in Figures 3 (e).

	Expected Ω	Fitted Ω (MHz)	LW (MHz)
$\nu_{\mathrm{RF}} = 17$ MHz	17 MHz	16.81 ± 0.05	3.17 ± 0.07
$\nu_{\mathrm{RF}} = 20$ MHz	20 MHz	19.85 ± 0.05	2.97 ± 0.08

Supplementary Table 10. Fitting parameters for the data shown in Figures 3 (f).

t_{SL} (μs)	Expected Ω	Fitted Ω (MHz)	LW (MHz)
0.1	16 MHz	16.50 ± 0.11	4.3 ± 0.2
0.5	16 MHz	15.94 ± 0.07	2.2 ± 0.1
1	16 MHz	15.80 ± 0.07	3.1 ± 0.1
3	16 MHz	16.0 ± 0.2	4.5 ± 0.3
6	16 MHz	15.7 ± 0.2	5.8 ± 0.3

Supplementary Note 9: Raw data

In order to obtain the true lineshape of the detected signal, background subtraction was performed on both the XY8-N and spinlock experiments depicted in Figures 3(b) and 3(e) of the main text. In the case of the XY8-2 experiment in Figure 3 (b), the background is an exponential decay since the V_B^- fluorescence contrast is monitored while the interpulse delay τ is swept (Supplementary Figure 15). For the spinlock experiments, a typical baseline is observed where the overall contrast increases with the spinlock amplitude [7] (see Supplementary Figure 16). This is due to the fact that, after the strong excitation of the V_B^- spins by the first $\pi/2$-pulse, the spinlock amplitude is incrementally increased, and the stronger the spinlock pulse becomes, the more efficiently it can lock the V_B^- spins on the transverse plane. Moreover, we notice a degrading intensity and broadening of the dip when $\Omega/(2\pi) \gtrsim 20\,\text{MHz}$, which is a sign of MW heating. Therefore, we avoided probing higher RF frequencies.

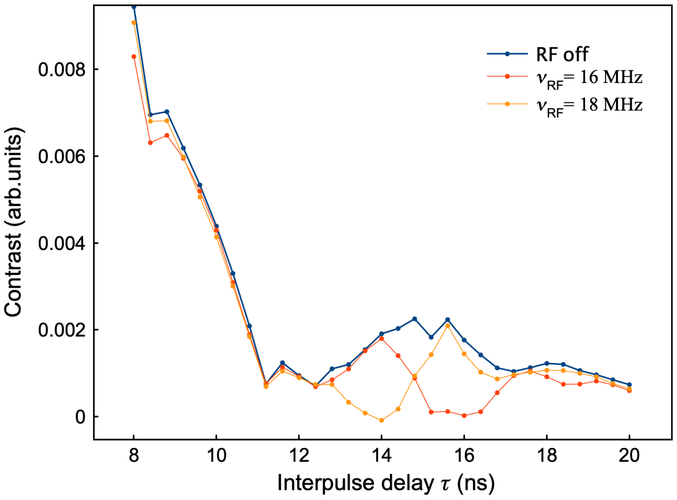

Supplementary Figure 15. Data of the XY8-2 experiments shown in Figure 3(b) before background subtraction.

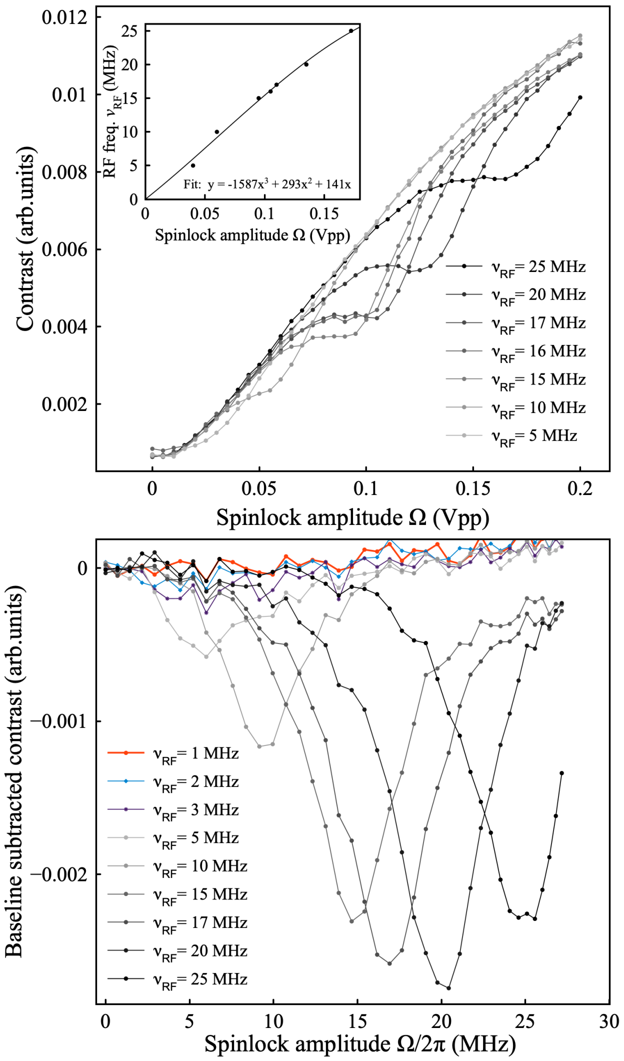

Supplementary Figure 16. Top: Raw data of the spinlock experiments before background subtraction. The datasets corresponding to $\nu_{\rm RF}$ =17 and 20 MHz are the ones reported in Figure 3 e). Inset: The amplitudes of the spinlock that resulted in depolarization dips when different $\nu_{\rm RF}$ were applied are graphed and a polynomial fit is performed. Bottom: Same data after background subtraction. Here, the experiments with $\nu_{\rm RF}$=1, 2 and 3 MHz are additionally shown.

[1] S. Stoll and A. Schweiger, EasySpin, a Comprehensive Software Package for Spectral Simulation and Analysis in EPR, Journal of Magnetic Resonance **178**, 42 (2006).

[2] V. Ivady, G. Barcza, G. Thiering, S. Li, H. Hamdi, J.-P. Chou, O. Legeza, and A. Gali, Ab Initio Theory of the Negatively Charged Boron Vacancy Qubit in Hexagonal Boron Nitride, npj Computational Materials **6**, 41 (2020).

[3] W. Liu, V. Ivdy, Z.-P. Li, Y.-Z. Yang, S. Yu, Y. Meng, Z.-A. Wang, N.-J. Guo, F.-F. Yan, Q. Li, J.-F. Wang, J.-S. Xu, X. Liu, Z.-Q. Zhou, Y. Dong, X.-D. Chen, F.-W. Sun, Y.-T. Wang, J.-S. Tang, A. Gali, C.-F. Li, and G.-C. Guo, Coherent Dynamics of Multi-Spin V_B^- Center in Hexagonal Boron Nitride, Nature Communications **13**, 5713 (2022).

[4] C. Degen, F. Reinhard, and P. Cappellaro, Quantum Sensing, Reviews of Modern Physics **89**, 035002 (2017).

[5] E. Abe and K. Sasaki, Tutorial: Magnetic resonance with nitrogen-vacancy centers in diamondmicrowave engineering, materials science, and magnetometry, Journal of Applied Physics **123**, 10.1063/1.5011231 (2018), _eprint: https://pubs.aip.org/aip/jap/article-pdf/doi/10.1063/1.5011231/13424065/161101_1_online.pdf.

[6] M. Loretz, T. Rosskopf, and C. L. Degen, Radio-Frequency Magnetometry Using a Single Electron Spin, Physical Review Letters **110**, 017602 (2013).

[7] R. Rizzato, F. Bruckmaier, K. Liu, S. Glaser, and D. Bucher, Polarization Transfer from Optically Pumped Ensembles of N-V Centers to Multinuclear Spin Baths, Physical Review Applied **17**, 024067 (2022).

[8] J. M. Boss, K. S. Cujia, J. Zopes, and C. L. Degen, Quantum Sensing with Arbitrary Frequency Resolution, Science **356**, 837 (2017).

[9] S. Schmitt, T. Gefen, F. M. Strner, T. Unden, G. Wolff, C. Mller, J. Scheuer, B. Naydenov, M. Markham, S. Pezzagna, J. Meijer, I. Schwarz, M. Plenio, A. Retzker, L. P. McGuinness, and F. Jelezko, Submillihertz Magnetic Spectroscopy Performed with a Nanoscale Quantum Sensor, Science **356**, 832 (2017).

[10] D. R. Glenn, D. B. Bucher, J. Lee, M. D. Lukin, H. Park, and R. L. Walsworth, High-Resolution Magnetic Resonance Spectroscopy Using a Solid-State Spin Sensor, Nature **555**, 351 (2018).

[11] J. E. Lang, T. Madhavan, J.-P. Tetienne, D. A. Broadway, L. T. Hall, T. Teraji, T. S. Monteiro, A. Stacey, and L. C. L. Hollenberg, Nonvanishing Effect of Detuning Errors in Dynamical-Decoupling-Based Quantum Sensing Experiments, Physical Review A **99**, 012110 (2019).

[12] A. M. Souza, G. A. lvarez, and D. Suter, Robust Dynamical Decoupling, Philosophical Transactions of the Royal Society A: Mathematical, Physical and Engineering Sciences **370**, 4748 (2012).

[13] D. Farfurnik, A. Jarmola, L. M. Pham, Z. H. Wang, V. V. Dobrovitski, R. L. Walsworth, D. Budker, and N. Bar-Gill, Optimizing a Dynamical Decoupling Protocol for Solid-State Electronic Spin Ensembles in Diamond, Physical Review B **92**, 060301 (2015).

[14] J. F. Barry, J. M. Schloss, E. Bauch, M. J. Turner, C. A. Hart, L. M. Pham, and R. L. Walsworth, Sensitivity Optimization for NV-Diamond Magnetometry, Reviews of Modern Physics **92**, 015004 (2020).

[15] E. V. Levine, M. J. Turner, P. Kehayias, C. A. Hart, N. Langellier, R. Trubko, D. R. Glenn, R. R. Fu, and R. L. Walsworth, Principles and Techniques of the Quantum Diamond Microscope, Nanophotonics **8**, 1945 (2019).

[16] R. Gong, G. He, X. Gao, P. Ju, Z. Liu, B. Ye, E. A. Henriksen, T. Li, and C. Zu, Coherent Dynamics of Strongly Interacting Electronic Spin Defects in Hexagonal Boron Nitride, arXiv:2210.11485 [quant-ph] 10.48550/arXiv.2210.11485 (2022).

[17] I. P. Radko, M. Boll, N. M. Israelsen, N. Raatz, J. Meijer, F. Jelezko, U. L. Andersen, and A. Huck, Determining the internal quantum efficiency of shallow-implanted nitrogen-vacancy defects in bulk diamond, Opt. Express **24**, 27715 (2016).

[18] W. Liu, N.-J. Guo, S. Yu, Y. Meng, Z.-P. Li, Y.-Z. Yang, Z.-A. Wang, X.-D. Zeng, L.-K. Xie, Q. Li, J.-F. Wang, J.-S. Xu, Y.-T. Wang, J.-S. Tang, C.-F. Li, and G.-C. Guo, Spin-Active Defects in Hexagonal Boron Nitride, Materials for Quantum Technology **2**, 032002 (2022).

[19] K. S. Liu, A. Henning, M. W. Heindl, R. D. Allert, J. D. Bartl, I. D. Sharp, R. Rizzato, and D. B. Bucher, Surface NMR using quantum sensors in diamond, Proceedings of the National Academy of Sciences **119** (2022).

[20] J. Henshaw, P. Kehayias, L. Basso, M. Jaris, R. Cong, M. Titze, T.-M. Lu, M. P. Lilly, and A. M. Mounce, Mitigation of nitrogen vacancy photoluminescence quenching from material integration for quantum sensing, Materials for Quantum Technology **3**, 035001 (2023).

[21] J. Henshaw, P. Kehayias, M. Saleh Ziabari, M. Titze, E. Morissette, K. Watanabe, T. Taniguchi, J. I. A. Li, V. M. Acosta, E. S. Bielejec, M. P. Lilly, and A. M. Mounce, Nanoscale solid-state nuclear quadrupole resonance spectroscopy using depth-optimized nitrogen-vacancy ensembles in diamond, Applied Physics Letters **120**, 174002 (2022).

[22] I. Schwartz, J. Rosskopf, S. Schmitt, B. Tratzmiller, Q. Chen, L. P. McGuinness, F. Jelezko, and M. B. Plenio, Blueprint for nanoscale NMR, Scientific Reports **9**, 6938 (2019).

[23] X. Gao, S. Vaidya, K. Li, P. Ju, B. Jiang, Z. Xu, A. E. Allcca Llacsahuanga, K. Shen, T. Taniguchi, K. Watanabe, S. A. Bhave, Y. P. Chen, Y. Ping, and T. Li, Nuclear Spin Polarization and Control in Hexagonal Boron Nitride, Nature Materials **21**, 1024 (2022).
